# Exercise training maintains cardiovascular health: signaling pathways involved and potential therapeutics

**DOI:** 10.1038/s41392-022-01153-1

**Published:** 2022-09-01

**Authors:** Huihua Chen, Chen Chen, Michail Spanos, Guoping Li, Rong Lu, Yihua Bei, Junjie Xiao

**Affiliations:** 1 School of Basic Medical Science, Shanghai University of Traditional Chinese Medicine, Shanghai, 201203 China; 2 Shanghai Engineering Research Center of Organ Repair, School of Medicine, Shanghai University, Shanghai, 200444 China; 3 Cardiac Regeneration and Ageing Lab, Institute of Cardiovascular Sciences, School of Life Science, Shanghai University, Shanghai, 200444 China; 4 Cardiovascular Division of the Massachusetts General Hospital and Harvard Medical School, Boston, MA 02114 USA

**Keywords:** Non-coding RNAs, Cardiology

## Abstract

Exercise training has been widely recognized as a healthy lifestyle as well as an effective non-drug therapeutic strategy for cardiovascular diseases (CVD). Functional and mechanistic studies that employ animal exercise models as well as observational and interventional cohort studies with human participants, have contributed considerably in delineating the essential signaling pathways by which exercise promotes cardiovascular fitness and health. First, this review summarizes the beneficial impact of exercise on multiple aspects of cardiovascular health. We then discuss in detail the signaling pathways mediating exercise’s benefits for cardiovascular health. The exercise-regulated signaling cascades have been shown to confer myocardial protection and drive systemic adaptations. The signaling molecules that are necessary for exercise-induced physiological cardiac hypertrophy have the potential to attenuate myocardial injury and reverse cardiac remodeling. Exercise-regulated noncoding RNAs and their associated signaling pathways are also discussed in detail for their roles and mechanisms in exercise-induced cardioprotective effects. Moreover, we address the exercise-mediated signaling pathways and molecules that can serve as potential therapeutic targets ranging from pharmacological approaches to gene therapies in CVD. We also discuss multiple factors that influence exercise’s effect and highlight the importance and need for further investigations regarding the exercise-regulated molecules as therapeutic targets and biomarkers for CVD as well as the cross talk between the heart and other tissues or organs during exercise. We conclude that a deep understanding of the signaling pathways involved in exercise’s benefits for cardiovascular health will undoubtedly contribute to the identification and development of novel therapeutic targets and strategies for CVD.

## Introduction

Cardiovascular disease (CVD) remains the leading cause of mortality worldwide.^1^ Data from a myriad of clinical and basic research studies have demonstrated the beneficial effects of exercise training on cardiovascular health, while physical inactivity has long been considered as a critical cardiovascular risk factor for the development of CVD.^2,3^ An accumulating body of cohort studies, systematic reviews, and meta-analyses have documented the beneficial effects of physical activity in reducing cardiovascular risk factors and risk of cardiovascular events.^4–7^ Moreover, appropriate physical activity also improves cardiorespiratory fitness and reduces all-cause mortality and CVD mortality.^8–13^ The 2018 Physical Activity Guidelines for Americans recommend at least 60 min of physical activity daily, for children and adolescents, and 150 min of moderate-intensity aerobic activity or 75 min of vigorous-intensity aerobic activity combined with 120 min of muscle-strengthening activities per week for adults.^14^ In fact, a recommendation for physical activity has been set in place and addresses the healthy population of all age groups. The 2019 UK Chief Medical Officers’ Physical Activity Guidelines recommend appropriate physical activity for the pregnant and post-partum women, older adults (≥65 years), and disabled people as well.^15^ The 2020 WHO guidelines recommend physical activity of moderate to vigorous intensity across all age groups and abilities.^16^ Owing to the fast-paced rhythm of living in modern societies, a vast majority of the population fails to meet the physical activity recommendation. However, increasing evidence indicates that even low levels of physical activity can be beneficial compared to inactivity.^16^ In fact, physical activity below the recommended daily level was still found to be associated with reduced all-cause mortality and extended life expectancy even for those at risk for CVD.^17^

In addition to the encouraging benefits of physical activity in the healthy population, exercise training has also been prescribed as medicine for different CVD.^18^ In clinical interventions, appropriate exercise training has been proven to enhance exercise capacity and cardiorespiratory fitness, reduce hospitalization, and improve life quality in patients with hypertension, coronary heart disease (CHD), cardiomyopathy, and heart failure (HF).^19^ Exercise plays a crucial role in both primary and secondary prevention of HF.^20^ Indeed, increasing research interest has been focused on the mechanisms of exercise’s benefits for cardiovascular health. Animal exercise models are useful tools to investigate and decipher the functional and molecular mechanisms of exercise-induced protection.^21^ Animal studies focused on cardiovascular health and diseases have demonstrated multifaceted beneficial effects of exercise and unraveled key mechanisms of exercise-induced cardiovascular protection.^22^ Accumulating evidence demonstrates that exercise is associated with reduced cardiovascular risk factors and cardiovascular events, and can exert protective effects on the myocardium through enhancing antioxidant capacity.^23^ The benefits of exercise in cardiovascular health can also be related to exercise-induced physiological cardiac hypertrophy, structural and functional vascular remodeling, as well as cardiac metabolic adaptations in mitochondrial function and glucose/lipid metabolism.^24^ Meanwhile, exercise induces systemic responses and influences other organs through extracellular vesicles and gut microbiota, which can also contribute to exercise-induced myocardial protection.^25–27^

In this Review, we will summarize the beneficial effects of exercise for cardiovascular health and then describe in detail the signaling pathways and their functional roles and mechanisms in exercise-induced cardioprotective effects. Next, we will address the exercise-mediated signaling pathways and molecules that can serve as potential therapeutic targets ranging from pharmacological approaches to gene therapies in CVD. The understandings of molecular pathway underlying exercise’s benefits for cardiovascular health will help promote the exercise training in both healthy population and patients with CVD, and develop novel therapeutic targets and strategies for CVD.

## Beneficial effects of exercise training for cardiovascular health

**Fig. 1:**
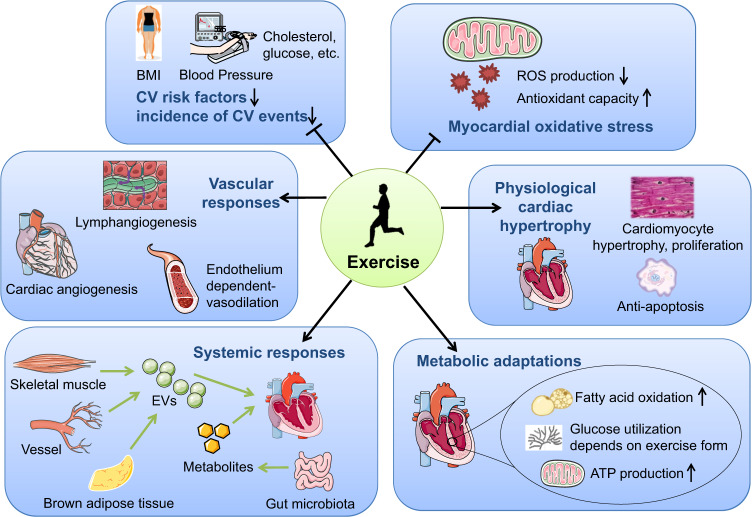
Beneficial effects of exercise training in cardiovascular health. Exercise training can exert beneficial effects for cardiovascular health through multiple aspects including reducing cardiovascular risk factors and cardiovascular events, reducing myocardial oxidative stress, promoting physiological cardiac hypertrophy, inducing vascular responses, cardiac metabolic adaptations, and systemic responses. BMI body mass index, CV cardiovascular, EVs extracellular vesicles, ROS reactive oxygen species

Exercise training can exert beneficial effects for cardiovascular health on multiple aspects. Physiologically, acute exercise increases the sympathetic activity while reduces the parasympathetic activity which releases catecholamines and subsequently increases heart rate and cardiac contractility. Meanwhile, exercise increases the blood flow back to the heart leading to increased left ventricle end-diastolic volume and force of contraction (Frank-Starling mechanism). Taken together, these physiological changes lead to increased stoke volume, heart rate, and cardiac output in response to acute exercise.^28^ A systematic review has recently reported that moderate-intensity of acute aerobic exercise is effective to reduce stress-induced blood pressure reactivity which may promote cardiovascular health.^29^ In response to chronic exercise, the heart and other tissues or organs will have both structural and functional adaptive changes which confer the beneficial effect of exercise. In the following part, we will discuss in detail the beneficial effects of chronic exercise training for cardiovascular health from multiple aspects (Fig. 1), including reducing cardiovascular risk factors and cardiovascular events, mitigating myocardial oxidative stress, promoting physiological cardiac hypertrophy and inducing vascular, cardiometabolic and systemic adaptations.

### Exercise reduces cardiovascular risk factors and cardiovascular events

The beneficial effect of exercise in reducing traditional cardiovascular risk factors, such as body mass index, total cholesterol, and blood pressure, are widely recognized.^3^ Moderate to vigorous physical activity has been shown to be associated with lower cardiometabolic risks, including blood pressure, glucose, insulin, and waist circumference, in US youth from 6 to 17 years.^30^ Meta-analysis of randomized controlled trials (RCTs) demonstrated beneficial effects of walking as an exercise intervention for cardiovascular risk factors such as body mass index, systolic and diastolic blood pressure, and fasting glucose in adults.^31^ Notably, exercise exerts favorable effects in reducing cardiovascular risk factors independently of the age group.^5,6,30,31^ Moreover, increasing number of prospective cohort studies and meta-analyses demonstrate that exercise reduces mortality and cardiovascular events. A large prospective cohort study across 17 countries reported that higher physical activity was associated with a lower risk of mortality and cardiovascular events.^32^ A Chinese population-based prospective cohort study in 10 areas across China showed that higher physical activity was associated with reduced risk of major vascular and coronary events.^33^ Physical activity was also shown to be associated with a lower incidence of cardiovascular events in old population after adjustment of cardiovascular risk factors.^34^ The correlation of the exercise type, intensity, and duration with the reduction of cardiovascular risk factors and cardiovascular events requires further investigation.

### Exercise reduces myocardial oxidative stress

Increased reactive oxygen species (ROS) production and altered antioxidant capacity represents a critical contributor to myocardial injury under pathological conditions and during the aging process.^35^ The heart contains a large number of mitochondria, which is an essential source of cellular ROS production.^36^ In addition, endothelial cells and neutrophils in the heart are also important sources of ROS production.^37^ Treadmill running rodent exercise studies demonstrated that exercise was effective to increase the protein abundance and/or activity of superoxide dismutase (SOD1 and SOD2), glutathione peroxidase, and catalase in the mitochondria fraction isolated from ventricular myocytes.^23^ Increased ROS production plays a key role in the development of myocardial ischemia/reperfusion (I/R) injury.^38^ Numerous studies have provided evidence supporting that exercise training is an effective intervention which reduces myocardial I/R injury.^39–41^ The protective effect of exercise against I/R injury was shown to be closely related to exercise-enhanced myocardial antioxidant capacity.^39^ In fact, exercise was shown to reduce the infarct size after myocardial I/R injury, an effect which was abolished by suppressing SOD2.^40,41^ In the early phase after I/R injury, ROS production was markedly lower in the hearts of exercised mice than sedentary mice.^42^ Mechanistically, exercise induced the endothelial nitric oxidase (eNOS)/nitric oxide (NO) and protein S‑nitrosylation pathway in the mitochondria, leading to reduced mitochondrial ROS production and mitochondrial permeability transition pore (mPTP) activation and thus, conveying cardioprotection against I/R injury.^42^ Further studies are needed to identify specific targets involved in exercise-induced protection against myocardial oxidative stress.

### Exercise promotes physiological cardiac hypertrophy

Cardiac hypertrophy is characterized by an increase in heart mass, and can be categorized as physiological or pathological cardiac hypertrophy.^43^ Pathological cardiac hypertrophy is commonly associated with diverse etiologies such as hypertension, arterial stenosis, myocardial infarction, and dilated cardiomyopathy, which progressively leads to cardiac remodeling and heart failure.^44,45^ Conversely, physiological cardiac hypertrophy is an adaptive and reversible cardiac growth that appears upon chronic exercise stimulation and exerts cardiac protective effects.^46,47^ Exercise-induced physiological cardiac hypertrophy exhibits multiple distinct characteristics that are different from pathological cardiac hypertrophy.^48^

From a structural and functional standpoint, endurance training such as swimming and long-distance running induces a volume overload which leads to eccentric cardiac hypertrophy, as characterized by a proportional change in ventricle wall thickness and chamber enlargement. On the contrary, strength training or pathological stimuli such as hypertension or aortic constriction causes concentric cardiac hypertrophy, as characterized by thickening of the left ventricle wall and a small reduction or no change in chamber size.^48^ Physiological cardiac hypertrophy does not develop myocardial apoptosis or cardiac fibrosis, and usually maintains normal or increased cardiac function. Furthermore, there is no expression change of fetal genes such as ANP, BNP, and β-MHC. Exercise-induced physiological cardiac hypertrophy exhibits adaptive protein synthesis and increased levels of fatty acid and glucose oxidation which help ensure cardiomyocyte growth and ATP production.^22,24^ Using animal exercise models of swimming, treadmill running, and voluntary wheel running, different research groups were able to show that exercise-induced cardiac hypertrophy is characterized by both hypertrophy and proliferation of cardiomyocytes.^47,49^ Exercise-induced physiological cardiac growth exhibits increased proliferation markers in cardiomyocytes such as EdU, Ki67, and phosphorylated histone H3 (pHH3).^47,49–51^ Current evidence indicates that exercise-induced cardiac cell proliferation, including cardiomyocyte proliferation, is necessary to mediate exercise’s benefit in preventing cardiac I/R injury.^52^ Recent evidence shows that exercise-induced cardiac protection is closely related to physiological hypertrophy, enhanced proliferation, and blunted apoptosis of cardiomyocytes.^22^

From a molecular standpoint, different signaling pathways are involved in physiological cardiac hypertrophy compared to pathological cardiac hypertrophy.^48^ Deciphering the different molecular mechanisms between physiological and pathological cardiac hypertrophy may provide novel therapeutic targets for CVD. It has been known that angiotensin II (Ang II), endothelin-1 (ET-1), norepinephrine, and their downstream signaling pathways (e.g., calcineurin, mitogen-activated protein kinase (MAPK), and calmodulin) contribute to the development of pathological cardiac hypertrophy.^48,53^ While the insulin growth factor-1/phosphoinositide-3 kinase/protein kinase B (IGF1/PI3K/Akt) pathway and the transcription factor CCAAT/enhancer binding protein β (C/EBPβ)/CITED4 pathway are canonical signaling pathways that have been well described for exercise-induced physiological cardiac hypertrophy. With the increasing interest and understanding of the epigenetics and post-transcriptional regulation, the noncoding RNA-regulated signaling pathways have been identified in the exercised heart.^37,46,47,51^ Notably, the regulatory molecules contributing to exercise-induced physiological cardiac hypertrophy have the potential to exert cardiovascular protective effects.^54^ The signaling pathways and molecular aspects of exercise-induced physiological cardiac hypertrophy and cardiovascular protection will be discussed later in detail (Table 1).

**Table 1:** Signaling pathways and molecules involved in exercise-induced cardiac hypertrophy and their contributions to cardioprotection

Target	Intervention	Model	Effect	Reference
IGF1/PI3K/AKT	CM-specific IGF1R TG mice	At baseline	Physiological cardiac hypertrophy through activating PI3K(p110α)	^107^
	CM-specific IGF1R KO mice	Swimming	Blunted physiological cardiac hypertrophy	^108^
	Cardiac constitutively active PI3K (caPI3K) TG mice and dominant-negative PI3K (dnPI3K) TG mice	At baseline	caPI3K TG mice with enlarged hearts and dnPI3K TG mice with smaller hearts	^109^
	CM-specific dnPI3K TG mice	Swimming	Blunted physiological cardiac hypertrophy	^110^
	AKT1 KO mice	Swimming	Blunted physiological cardiac hypertrophy	^116^
	Swimming mice compared to CM-specific caPI3K TG mice	DCM	Swimming exercise and caPI3K TG had similar protective effects against DCM	^118^
	CM-specific dnPI3K TG mice	Swimming and aortic banding-induced pathological hypertrophy	Anti-hypertrophic effect of exercise was attenuated in dnPI3K TG mice	^119^
C/EBPβ/CITED4	Mice heterozygous for C/EBPβ (C/EBPβ^+/−^)	At baseline or TAC-induced pathological hypertrophy	C/EBPβ^+/−^ phenocopied exercise-induced physiological cardiac growth with both CM hypertrophy and proliferation and prevented TAC-induced pathological hypertrophy	^49^
	CM-specific CITED4 TG mice	At baseline or cardiac I/R injury	CITED4 TG increased heart weight and CM size without influencing CM proliferation in vivo, and attenuated cardiac remodeling after I/R injury	^128^
	CM-specific CITED4 KO mice	Swimming or TAC model	CITED4 KO induced maladaptive cardiac responses to exercise, and aggravated TAC-induced pathological hypertrophy	^129^
miR-222	LNA-anti-miR-222 or CM-specific miR-222 TG mice	Swimming or cardiac I/R injury	Reducing miR-222 blunted physiological hypertrophy, and cardiac-specific miR-222 overexpression prevented cardiac remodeling after I/R injury	^47^
miR-17-3p	miR-17-3p agomiR or antagomiR	Swimming or cardiac I/R injury	miR-17-3p antagomiR blunted physiological hypertrophy, and miR-17-3p agomiR prevented cardiac remodeling after I/R injury	^186^
LncRNA CPhar	AAV9-mediated CPhar overexpression or inhibition	Swimming or cardiac I/R injury	AAV9-shCPhar blunted physiological hypertrophy, and AAV9-CPhar prevented cardiac remodeling after I/R injury	^51^
lncExACT1 and DCHS2	AAV9-mediated lncExACT1 overexpression and GapmeR-mediated lncExACT1 reduction	Running, cardiac I/R injury or TAC-induced pathological hypertrophy	Reducing lncExACT1 or DCHS2 induced physiological cardiac hypertrophy seen with exercise and prevented cardiac I/R injury and TAC-induced pathological hypertrophy, while increasing lncExACT1 or DCHS2 induced pathological cardiac hypertrophy	^46^

*CM* cardiomyocyte, *IGF1* insulin growth factor 1, *PI3K* phosphoinositide-3 kinase, *IGF1R* insulin growth factor 1 receptor, *TG* transgenic, *KO* knockout, *DCM* dilated cardiomyopathy, *C/EBPβ* CCAAT/enhancer binding protein β, *TAC* transaortic constriction, *I/R* ischemia/reperfusion, *miR* microRNA, *lncRNA* long noncoding RNA, *LNA* locked nucleic acid, *GapmeR* LNA antisense oligonucleotide

### Exercise induces vascular responses

Due to the increased demand for oxygen and energy during exercise, the cardiac vasculature responds in multiple ways. The accelerated heart rate and blood flow induce an increase in vascular shear stress, which further activates eNOS activity and promotes NO production from vascular endothelial cells.^55^ Exercise-induced activation of β3-adrenoceptor (β3-AR) and vascular endothelial growth factor receptor (VEGFR) also lead to NO production from vascular and cardiac endothelial cells.^56^ In the vasculature, endothelium-released NO diffuses into adjacent vascular smooth muscle cells and further leads to cGMP-dependent smooth muscle cell relaxation and vasodilation. The eNOS can be located in both endothelium and cardiomyocytes, and evidence indicates that exercise predominantly activates eNOS phosphorylation in coronary endothelium, but not in cardiomyocytes, to mediate the protective effect of exercise against I/R injury through improving endothelium-dependent coronary relaxation.^57^ In the myocardium, cardiac endothelium-derived NO or increased myocardial NO availability exerts multiple cardioprotective functions through modulating mitochondrial respiration, inhibiting β1-AR-induced contractility, and inducing cGMP-dependent relaxation of myocytes.^24,58,59^ On the other hand, cardiac endothelium-derived NO plays a key role in cardiac angiogenesis during exercise. It has been well established that pathological cardiac hypertrophy or myocardial ischemia injury are often associated with inadequate angiogenesis. Conversely, exercise training can induce proportional angiogenesis and dilation of the coronary vasculature to facilitate physiological cardiac hypertrophy and delivery of required oxygen and nutrients to the myocardium upon exercise.^60^ Furthermore, exercise can upregulate hypoxia-inducible factor (HIF)1α and peroxisome proliferator-activated receptor γ co-activator 1α (PGC1α) in the heart, leading to production of VEGF which is a crucial factor for angiogenesis.^61^ Exercise can also stimulate skeletal muscle-derived follistatin like-1 (FSTL1) which has been shown to promote endothelial cell proliferation and tube formation in vitro, to increase myocardial angiogenesis and to prevent cardiac remodeling and cardiac dysfunction after myocardial infarction in vivo.^62^

Equally to the blood vasculature, the lymphatic vasculature is also an essential component of the cardiovascular system.^63^ Cardiac lymphatic vessels play essential roles in the regulation of interstitial fluid homeostasis, immune cell trafficking, and lipid transport.^64^ Accumulating evidence has indicated that promoting lymphangiogenesis has beneficial effects in preventing a variety of CVD such as myocardial infarction, myocardial I/R injury, cardiac remodeling, and heart failure.^65–67^ In a mouse model of swimming exercise-induced physiological cardiac hypertrophy, cardiac lymphangiogenesis was significantly increased which was associated with VEGFR3 activation.^68^ The increase in cardiac lymphangiogenesis was found to be necessary for exercise-induced physiological cardiac hypertrophy and the increased proliferation markers in cardiomyocytes.^68^ Lymphatic endothelial cell-conditioned culture medium can promote both physiological hypertrophy and proliferation of cardiomyocytes in vitro.^68^ The secreted IGF1 and extracellular protein RELN by lymphatic endothelial cells may mediate the paracrine effect of lymphangiogenesis on exercise-induced physiological cardiac hypertrophy.^68^ Due to the beneficial effect of lymphangiogenesis in myocardial protection, it warrants additional studies to explore whether and how lymphangiogenesis contributes to exercised-induced cardiovascular protection.

### Exercise induces cardiac metabolic adaptations

Exercise is a physiological stressor which increases the hemodynamic function and energy production of the body. Cardiac mitochondria response dynamically to exercise to maintain cellular homeostasis and metabolic energy supply through the process of fusion, fission, and mitophagy.^69,70^ In response to acute exercise, increased cytosolic and mitochondrial Ca^2+^ promotes ATP production through increasing ATPase activity, dehydrogenase activity, and NADH oxidation.^71,72^ Meanwhile, acute exercise can rapidly induce mitochondrial fission to increase the number and surface area of mitochondria through activating dynamin-related protein 1 (Drp1).^73^ During chronic exercise, PGC1α and eNOS activations enhance mitochondrial biogenesis.^74,75^ In addition, exercise can also induce repeated cycle of mitochondrial fission and mitophagy to improve mitochondrial quality control.^76,77^

Glucose and fatty acids are the major sources of ATP production in the heart. Under physiological baseline conditions, fatty acid oxidative phosphorylation is responsible for 40–70% cardiac ATP production and glucose oxidative phosphorylation is responsible for 20%–30% cardiac ATP production.^78^ However, under pathological conditions such as myocardial infarction, pathological hypertrophy, and heart failure, a metabolic switch in energy substrate preference from fatty acid oxidation to glucose oxidation is undergone by the heart.^79^ This is partly due to the fact that fatty acid oxidation requires more oxygen and that the incomplete fatty acid β-oxidation causes accumulation of malondialdehyde (MDA) in the myocardium.^80,81^ However, glycolysis consumes less oxygen and its oxidative phosphorylation products, H_2_O and CO_2_, are non-toxic and safe for the heart.^82^

Compared to pathological conditions, exercise training induces distinct metabolic changes in the heart. Exercise increases cardiac workload and myocardial oxygen consumption, leading to increased rate of ATP production. These changes may increase myocardial carbohydrate and fatty acid catabolism.^83^ However, glucose uptake and oxidation can be diminished due to high levels of lactate or free fatty acids upon exercise training.^83,84^ At the same time, the increased lactate and free fatty acid levels will promote fatty acid uptake and utilization.^83,85^ Treadmill running exercise was found to be protective against myocardial I/R injury in female exercised rats, associated with increased fatty acid and glucose oxidation and reduced glycolysis.^86^ Swimming exercise was shown to attenuate acute myocardial infarction through positively regulating the genes involved in fatty acid metabolism and activating the PGC1α, PPARα, and PPARγ.^87^ In fact, different exercise type, intensity, and duration can either increase or decrease the uptake and utilization of glucose.^79^ In a mouse model that underwent swimming exercise, it was shown that exercise-induced adaptations lead to physiological cardiac hypertrophy and increased glycolysis, glucose oxidation, fatty acid oxidation, and ATP production.^88^ However, a study involving a mouse model of treadmill running exercise reported that glucose utilization through glycolysis was reduced immediately after a bout of acute exercise through reduced phosphofructokinase (PFK) activity, while the myocardial glycolysis was adaptively increased after chronic treadmill running.^89^ The study also revealed that low myocardial glycolytic activity was able to induce an increase in cardiac size, an adaptation similar to the exercise-induced physiological cardiac hypertrophy.^89^ The PPAR, PGC1α, AMPK signaling pathways have been shown to regulate the expressions of fatty acid oxidation genes, glycolytic genes, and mitochondrial biogenesis genes upon exercise.^90,91^ However, the periodicity and dynamic changes of cardiac metabolic adaptations upon exercise training and its contribution to physiological cardiac hypertrophy and exercise-induced cardiovascular protection require further investigation.

### Exercise induces systemic responses

Exercise is an effective modality that induces systemic responses in the body and promotes the release of various factors such as peptides, metabolites, mRNAs, and non-coding RNAs into the circulation, which then have the potential to participate in a systemic crosstalk and reach all organs and tissues, including the heart. Current research interest has been focused on the responses and functional roles of extracellular vesicles (EVs) such as small EVs (eg. exosomes of 30–150 nm) and large EVs (e.g. microparticles of 100–1000 nm) in the exercised body.^92,93^ EVs are small lipid double-layer membrane vesicles containing a variety of biological contents such as RNA, proteins, and lipids, which convey vital signals among cells and mediate intercellular communications.^94^ Many studies have investigated whether and how circulating EVs were changed upon different types of exercise. A systematic review has indicated that a single bout of exercise can induce a transient increase of circulating microparticles (CMPs) in healthy subjects, while chronically trained subjects may have an unchanged or low level of circulating CMPs due to their adaptations to exercise.^92^ The variable changes of circulating EVs after exercise can be influenced by multiple factors, such as exercise form (type, frequency, duration), time of blood draw, origin of EVs, as well as age and sex of exercised individuals.^92,95,96^

Emerging evidence has revealed the essential contribution of exercise-derived EVs to cardiovascular health. A 3-week swimming exercise was shown to increase the serum EV numbers in mice, and exercise-induced increase in circulating EV numbers further enhanced the protective effect of endogenous EVs against myocardial I/R injury and myocardial apoptosis.^97^ In another study using swimming exercised rat model, exercise-derived EVs were shown to have significant protective effect against myocardial I/R injury through their cargos containing miR-342-5p.^26^ Notably, endothelial cells were shown to be the major source of circulating exosomal miR-342-5p after exercise.^26^ In addition to the circulating EVs, brown adipose tissue was reported to be another source of exercise-derived cardioprotective EVs.^25^ Furthermore, small EVs secreted by brown adipose tissue mediated the protective effect of exercise against myocardial I/R injury through miR-125b-5p, miR-128-3p, and miR-30d-5p.^25^

In addition, exercise was also shown to influence the composition and metabolites of gut microbiome, which may play essential roles in regulating CVD.^98,99^ Both human and animal studies demonstrate that exercise can lead to higher richness and diversity of the gut microbiome.^98–100^ Treadmill running exercise was shown to be efficient in reducing myocardial injury post infarction in mice through rescuing gut microbial richness and bacterial community structure.^101^ Furthermore, two metabolites, 3-hydroxyphenylacetic acid (3-HPA) and 4-hydroxybenzoic acid (4-HBA), were identified to play essential roles in mediating beneficial effects against myocardial infarction through NRF2.^101^ Understanding the systemic responses that are activated upon exercise will lead to more considerate exercise interventions and therapeutic strategies for cardiovascular health.

## Signaling pathways mediating exercise’s benefits for cardiovascular health

### IGF1/PI3K/AKT signaling pathway

**Fig. 2:**
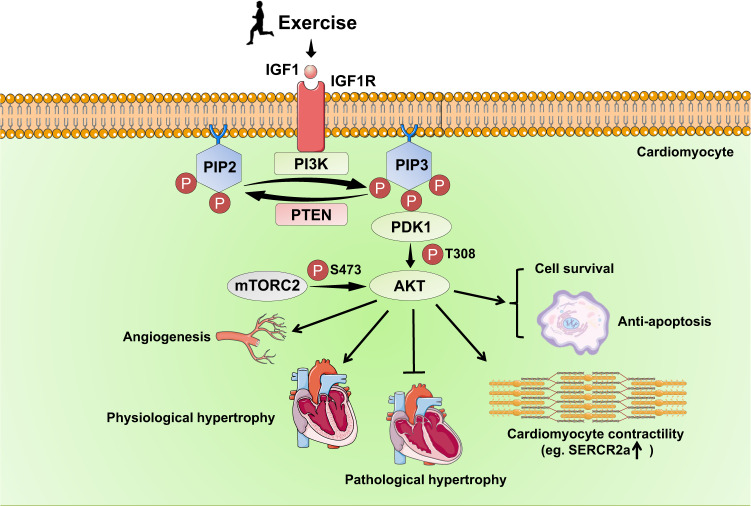
The IGF1/PI3K/AKT pathway in exercise-induced physiological cardiac hypertrophy and exercise-induced cardioprotection. Exercise induces IGF1 secretion, which activates IGF1R tyrosine kinase followed by recruitment of PI3K. The PI3K through converting PIP2 to PIP3 in the plasma membrane, further leads to recruitment of PDK1 and AKT to the plasma membrane and subsequent phosphorylation and activation of PDK1 and AKT. The activation of IGF1/PI3K/AKT is essential to mediate exercise-induced physiological cardiac hypertrophy through promoting cardiomyocyte growth in size. The IGF1/PI3K/AKT pathway also exerts cardioprotective effects through promoting cardiomyocyte contractility, enhancing survival while reducing apoptosis of cardiomyocytes, inhibiting pathological hypertrophy, and promoting angiogenesis. IGF1 insulin growth factor 1, IGFR1 insulin growth factor 1 receptor, PI3K phosphoinositide-3 kinase, PIP2 phosphatidylinositol 4,5-bisphosphate, PIP3 phosphatidylinositol 3,4,5-trisphosphate, PDK1 phosphoinositide dependent kinase 1, AKT protein kinase B, PTEN phosphatase and tensin homolog, mTORC2 mammalian target of rapamycin complex 2, SERCA2a sarco/endoplasmic reticulum Ca^2+^-ATPase 2a

The IGF1/PI3K/AKT signaling pathway has been well described for its essential role in mediating exercise-induced physiological cardiac hypertrophy and myocardial protection (Fig. 2). IGF1 is a hormone that can be released by both the liver and the heart. The increased cardiac and serum IGF1 levels have been observed in trained athletes with physiological cardiac hypertrophy.^102,103^ Exercise-induced IGF1 activates IGF1 receptor (IGF1R) tyrosine kinase, which can recruit the SH2 domain-containing protein such as the p85 regulatory subunit of Class IA PI3K.^104^ The lipid kinase Class IA PI3Ks contain different isoforms; one isoform which consists of a p85 regulatory subunit and a p110α catalytic subunit is the major PI3K isoform that plays important role in postnatal and exercise-induced cardiac growth as well as in exercise-induced cardiac protection.^105^ The p110α catalytic subunit of PI3K, through converting phosphatidylinositol 4,5-bisphosphate (PIP2) to phosphatidylinositol 3,4,5-trisphosphate (PIP3) in the plasma membrane, leads to recruitment of phosphoinositide-dependent kinase 1 (PDK1) and AKT to the plasma membrane. The binding of AKT with PIP3 leads to the conformational change and exposure of its phosphorylation sites Ser473 and Thr308. Finally, PIP3-induced phosphorylation of PDK1 leads to AKT phosphorylation at Thr308 and the mTOR complex 2 (mTORC2) leads to AKT phosphorylation at Ser473.^106^

A large number of studies using gene transgenic (TG) and knockout (KO) mice have revealed the critical involvement of IGF1/PI3K/AKT signaling pathway in exercise-induced physiological cardiac hypertrophy.^105^ Cardiac IGF1R overexpression induced physiological cardiac hypertrophy in mice, which was however blunted by cardiomyocyte-specific co-expression of a dominant negative PI3K(dnPI3K) p110α mutant.^107^ Cardiomyocyte-specific IGF1R knockout mice did not develop physiological cardiac hypertrophy after 5 weeks of swimming exercise.^108^ Genetic interventions of PI3K demonstrated that cardiac-constitutively active PI3K (caPI3K) TG mice had larger hearts, while cardiac expression of a dnPI3K p110α mutant resulted in smaller hearts.^109^ Moreover, mice with cardiac expression of a dnPI3K p110α mutant had blunted cardiac hypertrophic adaptations to swimming exercise.^110^ Despite a number of studies that reported somewhat conflicting results regarding the different cardiac phenotypes in response to increased AKT activity, it has currently been recognized that short-term appropriate AKT activation leads to physiological cardiac hypertrophy, while long-term AKT activation or AKT overexpression causes pathological cardiac hypertrophy, interstitial fibrosis, and even heart failure.^111–115^ However, AKT KO mice were resistant to develop physiological cardiac hypertrophy after swimming exercise, indicating an essential role of AKT activation in exercise-induced physiological cardiac hypertrophy.^116^ More recently, the transcription factor Forkhead box class O1 (FoxO1) was found to be required for exercise-induced physiological cardiac hypertrophy, but was not necessary in mediating PI3K activation-induced physiological hypertrophy in vivo.^117^

The IGF1/PI3K/AKT signaling pathway was not only investigated at baseline and in exercise-induced cardiac growth, but also explored in CVD with exercise intervention. Swimming exercise and cardiac-specific caPI3K overexpression had similar protective effects in a transgenic model of dilated cardiomyopathy (DCM).^118^ Exercise-induced activation of PI3K and AKT was shown to inhibit pathological stimulus-induced G protein-coupled receptor (GPCR) activation and its downstream MAPK activation such as extracellular signal-regulated kinase (ERK1/2), thus inhibiting pathological hypertrophy.^118^ Regular swimming exercise training prevented aortic banding-induced pathological cardiac hypertrophy, however, the protective effect of exercise was attenuated in banded cardiac-specific dnPI3K TG mice.^119^ On the contrary, cardiac-specific caPI3K expression was protective against pathological cardiac hypertrophy.^119^ These data suggest that PI3K is necessary for exercise-induced cardioprotection. The PI3K and/or AKT activation exhibits cardioprotective effects through promoting cardiomyocyte contractility, enhancing survival while inhibiting apoptosis of cardiomyocytes, and promoting angiogenesis, contributing further to exercise-induced cardioprotection.^120–123^ RNA sequencing and miRNA array revealed a group of mRNAs and miRNAs that were regulated in cardiac-specific caPI3K or dnPI3K TG mice, which can provide important information for further identifying downstream targets of the IGF1/PI3K/AKT signaling pathway in response to exercise.^124^

### C/EBPβ/CITED4 signaling pathway

The C/EBPβ belongs to the CCAAT enhancer-binding protein gene family which also includes C/EBPα, C/EBPδ, C/EBPε, C/EBPɣ, and C/EBPζ. The C/EBP family members share highly conserved basic-leucine zipper (bZIP) domain at the C-terminus which consists of basic amino-acid-rich DNA-binding region followed by leucine zipper, but differ a lot in sequence at the N-terminus.^125^ The C/EBPs act as important transcription factors, through forming either the homodimers or heterodimers with intrafamilial members or the heterodimers with other transcription factors containing bZIP domain or not, thus binding to specific gene promoters and regulating gene transcriptions.^126^ The C/EBPs have been well known for their pivotal roles in regulating many cell processes such as cell differentiation and proliferation.^127^

**Fig. 3:**
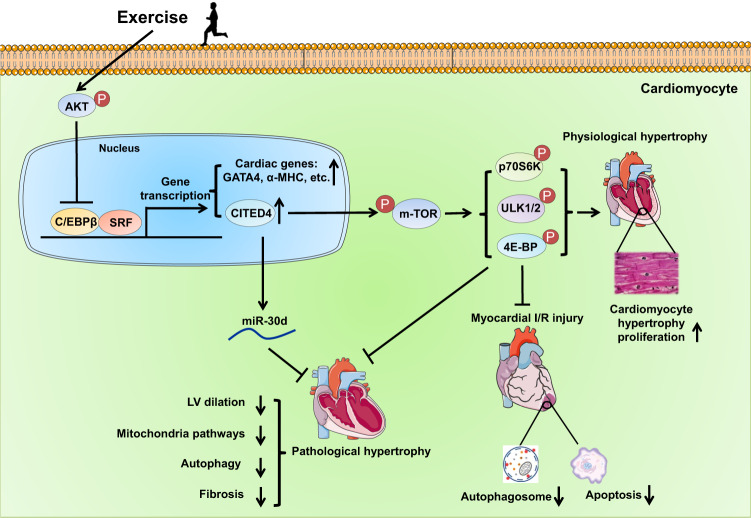
The C/EBPβ/CITED4 pathway in exercise-induced physiological cardiac hypertrophy and exercise-induced cardioprotection. Exercise leads to downregulation of the transcription factor C/EBPβ which is associated with increased AKT activity in the heart. The C/EBPβ interacts with SRF binding to the promoters of cardiac genes (e.g., GATA4 and α-MHC) and CITED4, leading to a negative transcriptional regulation of these genes. Increased CITED4 can act as a regulator of mTOR signaling with increased phosphorylation of p70S6K, ULK1/2, and 4E-BP, which mediates the beneficial effects of CITED4 in promoting physiological cardiac hypertrophy and preventing cardiac I/R injury. Increasing CITED4 is also able to prevent pathological hypertrophy through activating the mTOR pathway and upregulating anti-fibrotic miR-30d. C/EBPβ CCAAT/enhancer binding protein β, SRF serum response factor, CITED4 CBP/p300-interacting transactivator with ED-rich carboxyterminal domain 4, α-MHC α-myosin heavy chain, mTOR mammalian target of rapamycin, AKT protein kinase B, miR microRNA, LV left ventricle, CM cardiomyocyte, I/R ischemia-reperfusion

In recent years, increasing evidence has indicated that the C/EBPβ and its downstream target CBP/p300-interacting transactivator with ED-rich carboxyterminal domain 4 (CITED4) are essentially involved in exercise-induced physiological cardiac hypertrophy, and targeting the C/EBPβ/CITED4 signaling pathway is effective to prevent pathological cardiac hypertrophy and myocardial I/R injury (Fig. 3). Using RT-PCR-based screening against all transcriptional components, it has been identified that exercise training can downregulate C/EBPβ, while increasing CITED4 expression in the heart.^49^ Reducing C/EBPβ was sufficient to mimic exercise-induced physiological cardiac hypertrophic phenotype as evidenced by cardiomyocyte hypertrophy and proliferation.^49^ Meanwhile, reducing C/EBPβ promoted hypertrophic cell growth and proliferation of primary cardiomyocytes.^49^ C/EBPβ was further shown to interact with the transcription factor serum response factor (SRF) binding to the promoters of cardiac genes such as GATA4, α-MHC, and CITED4, thus leading to a negative transcriptional regulation of these genes; these genes also promoted hypertrophy and/or proliferation of primary cardiomyocytes.^49^ In addition to phenocopying the physiological cardiac growth, reducing C/EBPβ also protected against transaortic constriction (TAC)-induced pathological hypertrophy in mice.^49^

Cardiomyocyte-specific CITED4 expression was found to induce an increase in heart weight and cardiomyocyte size, while did not stimulate significant cardiomyocyte proliferation in the heart.^128^ Moreover, cardiac-specific CITED4 TG mice were resistant to cardiac I/R injury as evidenced by improved cardiac function, survival, and reduced cardiac fibrosis in the remodeling phase.^128^ Molecular analysis further revealed that CITED4 acted as a regulator of mTOR signaling leading to the activation of mTOR Complex 1 (mTORC1) including increased phosphorylation of p70S6K, ULK1/2, and 4E-BP, which mediated the effects of CITED4 in promoting cardiomyocyte growth in size and proliferation and in preventing cardiac I/R injury.^128^ On the contrary, cardiac-specific CITED4 KO mice had maladaptive cardiac responses to exercise as evidenced by ventricular dilation, reduced cardiac function, and upregulated pathological and fibrosis-associated genes.^129^ Moreover, cardiac-specific CITED4 KO mice manifested aggravated pathological hypertrophy, cardiac fibrosis, and cardiac dysfunction in TAC-induced pathological hypertrophy.^129^ Collectively, these data suggest that reduction of CITED4 causes deleterious effects in both physiological and pathological hypertrophy, which was found to be associated with impaired mitochondrial pathways, reduced mTORC1 activity, and downregulated anti-fibrotic miR-30d.^129^ Interestingly, C/EBPβ was negatively regulated by AKT activity in cardiomyocytes and contributed to AKT-regulated gene expression as seen with exercise training, which acts as connection between the C/EBPβ/CITED4 signaling and the IGF1/PI3K/AKT pathway in exercised-heart.^49^

### PPAR, PGC1α, AMPK signaling network

Exercise training efficiently induces mitochondria and energy metabolic changes to meet the increased demand of oxygen and energy in the heart. The peroxisome proliferator-activated receptor α (PPARα), being part of the ligand-activated nuclear receptor superfamily, is highly expressed in cardiac myocytes and can be activated by exercise.^130^ Compared to PPARα, PPARγ is expressed at lower level in the cardiac myocytes, and its expression and activity can be influenced by exercise in heart tissues and in extracardiac tissues (e.g., skeletal muscle) as well.^131,132^ PPARα can interact with the retinoid X receptor (RXR) to promote the transcription of target genes such as fatty acid transport protein 1 (FATP1), carnitine palmitoyltransferase I (CPTI), and medium-chain acyl-CoA dehydrogenase (MCAD) that contribute to fatty acid utilization including fatty acid uptake, thioesterification to fatty acyl-CoA, transport to mitochondria, and mitochondrial β-oxidation.^133,134^ These gene regulations may improve fatty acid oxidation while reduce free fatty acid accumulation in the myocardium upon injury.

**Fig. 4:**
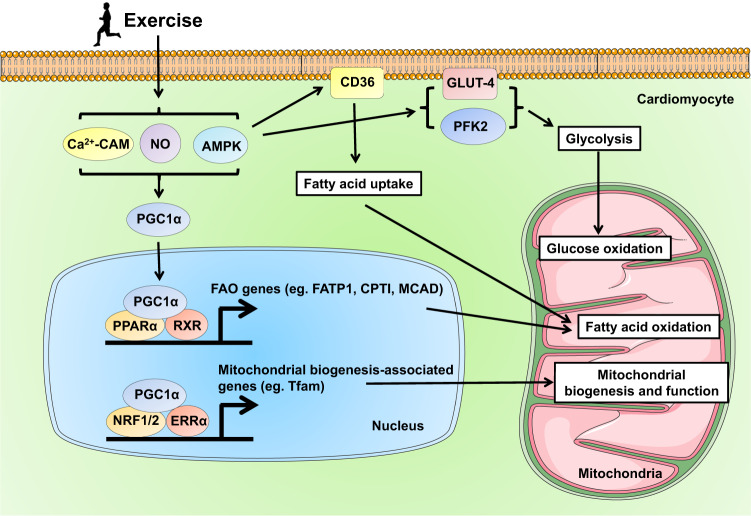
The PPAR, PGC1α, AMPK signaling network in exercise-induced metabolic changes and exercise-induced cardioprotection. Exercise triggers an increase in NO production, Ca^2+^-Calmodulin kinase interaction, and AMPK activation, leading to PGC1α upregulation and activation in cardiac muscle cells. The PGC1α may form transcriptional regulatory complex with PPARα and RXR, or coactivate other transcriptional partners such as NRF-1/2 and ERR, to promote downstream transcription of genes involved in fatty acid oxidation and mitochondrial biogenesis/energy metabolism, respectively. Exercise-induced AMPK may modulate glucose uptake, glycolysis and glucose oxidation, and may modulate fatty acid utilization including fatty acid uptake, translocation to mitochondrial, and fatty acid oxidation as well. PPARα peroxisome proliferator-activated receptor α, PGC1α peroxisome proliferator-activated receptor-alpha coactivator 1α, AMPK adenosine monophosphate-activated protein kinase, CAM calmodulin, NO nitric oxide, RXR retinoid X receptor, NRF1/2 nuclear respiratory factor-1 and 2, ERRα estrogen-related receptor α, FAO fatty acid oxidation, FATP1 fatty acid transport protein 1, CPTI carnitine palmitoyltransferase I, MCAD medium-chain acyl-CoA dehydrogenase, Tfam nuclear-encoded mitochondrial transcription factor A, PFK2 6-phosphofructo-2-kinase

The PGC1α, acting as an important transcriptional coactivator, can interact with other transcription factors and regulate target gene transcription. It has been recognized that exercise training triggers an increase in NO production, Ca^2+^-calmodulin kinase interaction, and adenosine monophosphate-activated protein kinase (AMPK) activation, which leads to PGC1α upregulation and activation in both skeletal and cardiac muscle cells.^135–138^ The PGC1α is a central transcriptional coactivator that links physiological exercise stimulus and metabolic changes in the heart (Fig. 4). The PGC1α/PPARα/RXR transcriptional regulatory complex promotes downstream transcription of genes involved in fatty acid oxidation pathway.^139^ In addition to regulating fatty acid metabolism, PGC1α is a master regulator of mitochondrial biogenesis and energy metabolism.^139^ The PGC1α can coactivate other transcriptional partners, such as nuclear respiratory factor-1 and 2 (NRF-1/2) and estrogen-related receptor (ERR) which activates transcription of the genes involved in mitochondrial biogenesis (e.g., nuclear-encoded mitochondrial transcription factor A, Tfam) and energy metabolism including tricarboxylic acid (TCA) cycle and oxidative phosphorylation.^140,141^

In addition, PGC1α can be activated by AMPK upon exercise training. AMPK is a serine/threonine kinase that can be activated by exercise stimulus associated with a decreased ATP/AMP ratio.^142,143^ The increased AMPK phosphorylation at Thr172 promotes glucose transport and glycolysis by regulating GLUT4 (translocation to cell membrane in myocardium and skeletal muscle) and 6-phosphofructo-2-kinase (PFK2).^144,145^ AMPK may also modulate fatty acid uptake, translocation to the mitochondrial, and fatty acid oxidation through regulating acetyl-CoA carboxylase (ACC), CD36, and malonyl-CoA decarboxylase (MCD).^144,146^ AMPK, through activating PGC1α, also contributes to mitochondrial biogenesis.^147^

Increasing evidence has demonstrated the protective effect of exercise against myocardial injuries through regulating the metabolic pathways. A 3-week swimming exercise regimen was shown to be protective and attenuate acute myocardial I/R injury in mice via the upregulation of PPARα, PPARγ, and PGC1α and regulation of the genes involved in fatty acid and glucose metabolism and mitochondrial biogenesis.^87^ Moreover, a 15-week treadmill running regimen was effective in preventing diabetic cardiomyopathy in db/db mice and was associated with improved mitochondrial biogenesis and activated PGC1α.^148^ In a streptozotocin/high-fat diet mouse model of diabetic cardiomyopathy, treadmill running also attenuated diabetic cardiomyopathy at least in part through improving PGC1α- and AMPK-dependent mitochondrial function.^149^ Thus, the PPAR, PGC1α, and AMPK signaling forms a complex network mediating the cardiac metabolic changes in response to exercise and contributes to exercise-induced cardioprotective effects.

### eNOS/NO signaling pathway

**Fig. 5:**
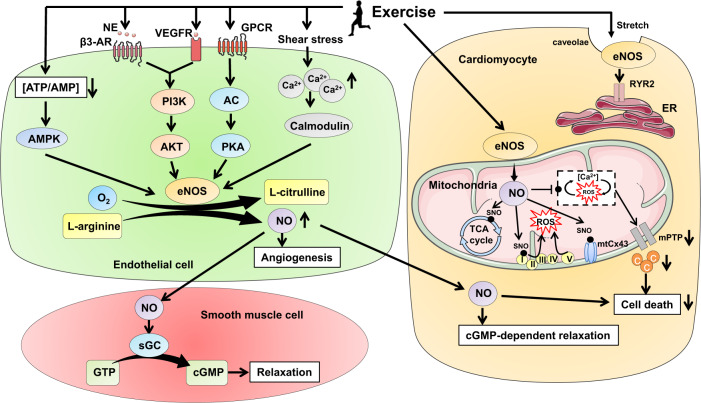
The eNOS/NO signaling pathway in exercise-induced vascular adaptations and exercise-induced cardioprotection. Exercise can activate the eNOS/NO signaling pathway through either calcium-dependent or -independent ways. Exercise-induced shear stress leads to an increase of intracellular Ca^2+^ within endothelial cells, which then interacts with calmodulin and activates eNOS and NO generation. Exercise can also activate the PI3K/AKT, AMPK, and PKA signaling pathways and subsequently increase eNOS phosphorylation and NO generation. The NO promotes angiogenesis or diffuses into adjacent vascular smooth muscle cells or cardiac muscle cells to induce cGMP-dependent relaxation. In cardiac muscle cells, exercise promotes the association of eNOS to the mitochondrial membrane, leading to increased NO bioavailability and S-nitrosylation of proteins involved in mitochondrial respiratory function, electron transport, and mPTP opening. The NO can further inhibit ROS and Ca^2+^ interaction in the mitochondria and then protect cardiomyocytes from cell death. Meanwhile, the eNOS located in the caveolae within cardiac myocytes can also be activated by exercise-associated myocardial stress to facilitate the opening of sarcoplasmic reticulum RyR2. eNOS endothelial nitric oxide synthase, NO nitric oxide, NE norepinephrine, β3-AR β3-adrenoceptor, VEGFR vascular endothelial growth factor receptor, PI3K phosphoinositide-3 kinase, AKT protein kinase B, AMPK adenosine monophosphate-activated protein kinase, GPCR G protein-coupled receptor, AC adenylate cyclase, PKA protein kinase A, sGC soluble guanylyl cyclase, cGMP cyclic guanosine monophosphate, SNO N-nitrosylation, ROS reactive oxygen species, mPTP mitochondrial permeability transition pore, c cytochrome c, ER endoplasmic reticulum, RyR2 reticulum ryanodine receptor type 2

Modulation of the eNOS/NO signaling contributes to exercise-induced vascular adaptations and cardioprotective effects (Fig. 5). Exercise-induced vascular shear stress is an efficient stimulus to increase intracellular Ca^2+^.^150^ The intracellular Ca^2+^, through interacting with calmodulin, leads to the dissociation of eNOS from caveolin and then activates eNOS to generate NO from L-Arginine in endothelial cells.^55^ Apart from the calcium-dependent eNOS activation, exercise also stimulates hormone and growth factor secretions such as norepinephrine (NE) and vascular endothelial growth factor (VEGF), which may bind to β3-AR and VEGFR in endothelial cells and further activate PI3K/AKT signaling and subsequent eNOS phosphorylation.^151,152^ Exercise-induced activation of β3-AR was shown to be necessary to activate eNOS phosphorylation and NO generation in the heart that was protective against myocardial I/R injury.^153^ In addition, exercise can lead to eNOS phosphorylation through protein kinase A (PKA) and AMPK.^154,155^ The phosphorylation of eNOS at Ser1177, Ser 615, and/or Ser633 leads to an increase in eNOS activity and NO production.^151,156,157^ Furthermore, exercise-induced myocardial stretch can activate eNOS that is located in the caveolae within cardiac myocytes, which then facilitates the opening of sarcoplasmic reticulum ryanodine receptor type 2 (RyR2).^158^

Exercise-induced NO in endothelial cells will diffuse into vascular smooth muscle cells, where it activates soluble guanylyl cyclase (sGC) to induce catalytic conversion of GTP to cyclic guanosine monophosphate (cGMP); the latter will further activate protein kinase G (PKG) and lead to relaxation of smooth muscle cells.^159,160^ The eNOS can be expressed in both coronary endothelium and cardiomyocytes in the heart, with the majority being in endothelial cells.^161^ Recent studies have shown that exercise increases eNOS phosphorylation at Ser1177 and protein S-nitrosylation mainly in endothelial cells but not in cardiomyocytes.^57^ The isolated segments of left coronary arteries from exercised rats showed enhanced endothelium-dependent vasorelaxation to acetylcholine (Ach) in vitro, and the I/R-induced alterations in endothelium-dependent vasodilation was also improved in the coronary artery segments isolated from exercised group.^57^ While the protective effect of exercise against myocardial I/R injury was attenuated in endothelium-inactivated rats, suggesting an essential contribution of coronary endothelium to exercise-induced cardioprotective effects.^57^ A paracrine effect of diffused NO into cardiomyocytes may also protect the myocardium from I/R injury with reduced lactate dehydrogenase (LDH) activity.^162^ In addition, it was reported that exercise can increase the association of eNOS with the mitochondria within the cardiac myocytes, thus increasing the bioavailability of NO and subsequent S-nitrosylation of the proteins involved in mitochondrial respiratory function, electron transport, and mPTP opening.^42^ The increased NO bioavailability in mitochondria has also the potential to blunt the interplay between ROS production and Ca^2+^ overload and then protect cardiomyocytes from cell death.^42^

Exercise-induced activation of the eNOS/NO pathway also exerts cardiovascular protective effects through modulating angiogenesis.^163,164^ The aging-related reduction of capillary density in the heart was improved by 8 weeks of swimming exercise in old rats, which was attributed to the increased VEGF angiogenic signaling cascade and subsequent phosphorylation of AKT and eNOS.^165^ Regular exercise training can upregulate miR-126 that targets phosphoinositol-3 kinase regulatory subunit 2 (PI3KR2), a negative regulator of PI3K activity, thus leading to an activation of PI3K/AKT/eNOS signaling that mediates the angiogenetic effect of miR-126 in exercised heart.^166^ MiR-126 also downregulates the target gene Sprouty-related protein 1 (SPRED1) which then activates the Raf-1/ERK1/2 signaling contributing to angiogenesis.^166^ Exercise has been shown to trigger an upregulation of HIF1α that acts as an upstream regulator of miR-126, which then targets PI3KR2 and SPRED1 and promotes angiogenesis through activating PI3K/AKT/eNOS signaling in myocardial infarction rats.^167^ Exercise training has also been known to increase spleen-derived and circulating endothelial progenitor cells (EPCs) via eNOS and VEGF upregulation, which prevents carotid artery injury through promoting angiogenesis.^168^ Collectively, the eNOS/NO signaling pathway mediates cardioprotective effects through acting on different types of cells, including endothelial cells, cardiomyocytes, and smooth muscle cells. To define the cardioprotective effects of different types of exercise training attributed to the eNOS/NO signaling pathway, further investigation is required.

### Noncoding RNAs and their regulated signaling pathways

Noncoding RNAs are key epigenetic regulators of gene expression that account for the regulation of cell homeostasis and control a wide range of diseases.^169,170^ With the development of next-generation sequencing technologies, different types of noncoding RNAs, such as microRNA (miRNA), long-noncoding RNA (lncRNA), and circular RNA (circRNA), have been increasingly identified and explored in different cardiovascular physiological and pathophysiological processes.^171–175^ Exercise training was effective to induce miR-29a/c expression in the heart which was associated with downregulated collagen gene expressions.^176^ Some other noncoding RNAs and their potential downstream targets were identified and proposed in the heart in response to exercise training, such as miR-1 and miR-214 (targeting NCX and SERCA2a),^177^ miR-27a/b and miR-143 (targeting angiotensin-converting enzyme-Angiotensin II genes),^178^ miR-208a/b (targeting Med13, Purβ, Sox6, HP1β, and SP3), and miR-21, miR-144, miR-145, miR-124 (targeting PI3K/AKT signaling).^179^

**Fig. 6:**
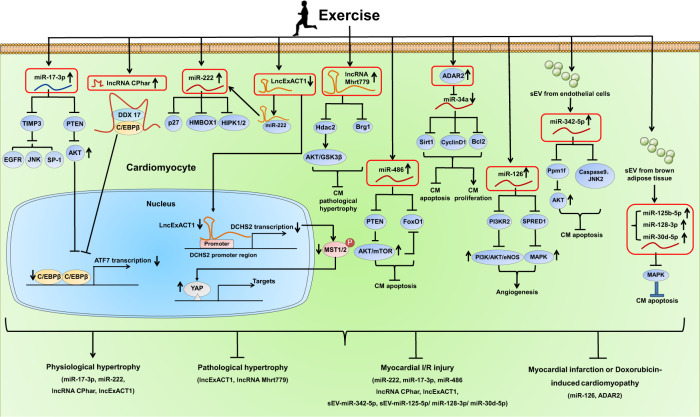
Noncoding RNAs and their regulated pathways in exercise-induced physiological cardiac hypertrophy and cardioprotection. Exercise upregulates miR-222 (targeting p27, Hmbox1, HIPK1/2) and miR-17-3p (targeting PTEN and TIMP3) in the heart. Exercise also upregulates lncRNA CPhar while downregulates lncExACT1 in the heart. LncRNA CPhar acts as a binding partner of DDX17 which sequesters C/EBPβ, thus leading to reduced transcription of ATF7. LncExACT1 is able to bind miR-222, while exercise-induced downregulation of lncExACT1 frees more miR-222 which contributes to exercise-induced physiological cardiac hypertrophy. LncExACT1 also positively regulates its closest protein-coding gene DCHS2 and reduced lncExACT1 activates YAP. Targeting these noncoding RNAs contributes to exercise-induced physiological cardiac hypertrophy and exerts protective effects against myocardial I/R injury and pathological cardiac hypertrophy. Exercise also regulates other noncoding RNAs including lncRNA Mhrt779, miR-486, ADAR2-regualted miR-34a, and miR-126, and induces endothelial cell-derived small extracellular vesicles (sEV) containing miR-342-5p and brown adipose tissue-derived sEV containing miR-125b-5p, miR-128-3p, and miR-30d-5p in the heart, which protect against myocardial injury and cardiac remodeling

The noncoding RNAs that have been found regulated in exercised heart may have functional roles in mediating exercise-induced cardioprotective effects (Fig. 6). Exercise-induced upregulation of miR-126 promoted angiogenesis and protected the heart from myocardial I/R injury and myocardial infarction through targeting PI3KR2 and SPRED1.^166,167^ Muscle-enriched miR-486 was induced by exercise and was necessary to mediate the beneficial effect of exercise in reducing I/R injury and myocardial apoptosis through targeting PTEN and FoxO1.^180^ However, circulating extracellular vesicles depleted of miR-486 lose their protective effect against cardiomyocyte apoptosis.^181^ LncRNA Mhrt779 was upregulated after 3 weeks of swimming exercise and continued to increase even at 1 week after swimming cessation; increased Mhrt779 was responsible for the beneficial effect of exercise hypertrophic preconditioning that prevented pathological cardiac hypertrophy, through binding to Brg1 and regulating the histone deacetylase 2 (HDAC2)/AKT/glycogen synthase kinase 3β (GSK3β) signaling pathway.^182^ Exercise training leads to upregulation of ADAR2, a member of the adenosine deaminases acting on RNA, which converts adenosine to inosine (A to I).^37^ Exercise-induced ADAR2 was revealed to negatively regulate miR-34a through A to I editing of pri-miR-34a.^37^ Increasing ADAR2 or inhibiting miR-34a promoted the proliferation and at the same time, inhibited apoptosis of cardiomyocytes, without influencing cardiomyocyte size.^37^ Notably, increasing ADAR2 was able to prevent myocardial infarction or doxorubicin-induced cardiomyopathy through downregulating miR-34a and modulating its target genes Sirt1, Cyclin D1, and Bcl2.^37^ Interestingly, the key transcription factor C/EBPβ regulated by exercise could bind to the promoter region of ADAR2 and negatively regulate its expression.^37^

It has been well-established that exercise can also modulate the circulating noncoding RNAs and extracellular vesicle-associated noncoding RNAs which may in turn, influence the development of CVD or serve as potential biomarkers for exercise adaptations and CVD.^183–185^ Exercise-induced exosomal miR-342-5p mainly derived from the endothelial cells conveyed cardioprotective effect against myocardial I/R injury through targeting Caspase 9, Jnk2, and Ppm1f, which reduced apoptosis while promoted survival of cardiomyocytes.^26^ Exercise also induced brown adipose tissue-derived small EVs containing a group of miRNAs (miR-125b-5p, miR-128-3p, and miR-30d-5p), which protected against myocardial I/R injury through targeting the molecules (e.g., MAP3K5, MAP2K7, AND MAP2K4) involved in the pro-apoptotic MAPK signaling pathway.^25^ These studies indicate that extracardiac tissue-derived EVs and their contained cargos may serve as alternative candidates to mediate exercise-associated cardioprotection.

In recent years, some noncoding RNAs that contribute to exercise-induced physiological cardiac hypertrophy have been shown to exert protective effects against myocardial I/R injury and cardiac remodeling.^46,47,51,186^ MiRNAs are a large group of highly conserved small noncoding RNAs of 18 to 25 nucleotide long, which negatively regulate downstream target genes by inducing transcript degradation or inhibiting protein translation.^187^ Based on miRNA array and RT-qPCR, miR-222 was identified to be upregulated in the heart in both swimming and voluntary wheel running exercise models.^47^ Although cardiomyocyte-specific increase of miR-222 was not sufficient to recapitulate the exercised heart phenotype, reducing miR-222 markedly attenuated exercise-induced physiological cardiac hypertrophy in vivo.^47^ MiR-222 promoted the physiological growth in size and proliferation, while inhibited apoptosis of primary cardiomyocytes.^47^ Interestingly, cardiac-specific expression of miR-222 was effective to increase proliferation markers of cardiomyocytes, reduce myocardial apoptosis, and prevent cardiac remodeling and dysfunction after I/R injury.^47^ The P27, Hmbox1, and HIPK1/2 were identified as target genes of miR-222.^47^ The serine/threonine protein kinase HIPK2 was further demonstrated to be upregulated in exercised heart.^188^ Interestingly, HIPK2 knockout or HIPK2 inhibitor, and miR-222 overexpression were found to be protective against myocardial infarction in mice.^188^ Mechanistically, inhibiting HIPK2 reduced apoptosis of primary cardiomyocytes and human embryonic stem cell-derived cardiomyocytes (hESC-CMs) through inhibiting P53.^188^ Since increasing miR-222 alone was not sufficient to induce physiological cardiac hypertrophy, other noncoding RNAs may have the potential to contribute to exercise-induced cardiac hypertrophy. MiR-17-3p was further identified as a key miRNA that mediates exercise-induced physiological cardiac hypertrophy and protects the heart from I/R injury through targeting TIMP3 and PTEN.^186^ Indeed, noncoding RNAs that contribute to exercise-induced physiological hypertrophy may be potential targets to mitigate pathological hypertrophy.^54^ Exercise-regulated molecules including noncoding RNAs may regulate multiple pathways and mechanisms (pro-hypertrophy, pro-proliferation, anti-apoptosis) that converge in conferring cardiovascular protective effects.^22^

LncRNAs are a heterogenous group of noncoding RNAs whose transcript size is more than 200 nucleotides.^189^ LncRNAs can be located in the nucleus or cytoplasm, thus regulating gene expressions and chromatin status in cis or in trans, modulating nuclear structure and organization, and interacting with numerous proteins and/or RNA molecules.^189^ A growing number of lncRNAs has been unraveled to play pivotal roles in myocardial injury and CVD such as pathological cardiac hypertrophy, myocardial infarction, cardiac fibrosis, and heart failure.^190^ Recently, lncRNAs were shown to contribute to exercise-induced physiological cardiac hypertrophy and cardioprotective effects.^189^ Based on lncRNA microarray, lncRNA CPhar was found to be markedly upregulated in the hearts of swimming exercised mice, while downregulated in TAC-induced pathological cardiac hypertrophy.^51^ CPhar had pro-hypertrophic, pro-proliferative, and anti-apoptotic effects in primary cardiomyocytes in vitro, and was necessary to mediate exercise-induced physiological cardiac hypertrophy in vivo.^51^ Moreover, increasing CPhar was protective against myocardial I/R injury and cardiac dysfunction in mice.^51^ CPhar acts as a binding partner of DDX17 which sequesters C/EBPβ, a key transcription factor involved in exercise-induced physiological cardiac hypertrophy, thus leading to reduced transcription of ATF7.^51^ More recently, lncRNA lncExACT1 was identified to be decreased in exercised heart while increased in pathological cardiac hypertrophy and heart failure.^46^ Of note, increasing lncExACT1 induced pathological hypertrophy, while reducing lncExACT1 induced physiological hypertrophy phenotypes seen with exercise training (myocardial hypertrophy, increased proliferation markers of cardiomyocytes) and improved cardiac function in vivo.^46^ LncExACT1 is able to bind miR-222, while exercise-induced downregulation of lncExACT1 releases more miR-222 which in turn contributes to exercise-induced physiological cardiac hypertrophy.^46^ LncExACT1 also positively regulates its closest protein-coding gene DCHS2 and reduced lncExACT1 activates YAP, which mediates the effect of lncExACT1 on cardiomyocyte growth and proliferation.^46^ Moreover, modulating DCHS2 was sufficient to regulate physiological and pathological cardiac hypertrophy in vivo.^46^ These studies reinforce the message that lncRNAs and their regulated signaling pathways involved in exercise-induced physiological cardiac hypertrophy may be potential therapeutic targets for CVD.^191^

## Potential therapeutic strategies based on exercise-regulated signaling pathways

It has been widely accepted that exercise is medicine and may serve as the real polypill to prevent and treat a variety of diseases including CVD.^192^ Physical inactivity is associated with 30% of ischemic heart disease burden, while exercise has protective effects in reducing cardiovascular risk factors and major cardiovascular events. For example, dynamic endurance exercise showed similar effect in reducing total cholesterol and low-density lipoprotein (LDL) cholesterol when compared to drug interventions.^193^ For those who can exercise appropriately, exercise is one of the most cost-effective interventions for cardiovascular health. However, for those who cannot exercise efficiently, exercise mimetics which specifically mimic some aspects of exercise-induced cardiovascular responses will alternatively bring about beneficial effects.^194^

The increasing knowledge of exercise-regulated signaling pathways will lead to the investigation of potential therapeutic targets and strategies that have protective effects against CVD in experimental models. Numerous studies have shown that activating the IGF1/PI3K/AKT signaling pathway has beneficial effects in a wide range of CVD such as myocardial infarction, myocardial I/R injury, diabetic cardiomyopathy, atrial fibrillation, and heart failure.^124,195–198^ These studies have used global or cardiac-specific genetically modified murine models targeting IGF1R, PI3K, and AKT.^107–110,116^ However, most of the genetically modified animals have undergone regulated gene expression or activity before the onset of CVD, indicating that the cardiovascular protective effects were predominately preventive. On the other hand, long-term activation of the IGF1/PI3K/AKT pathway may have detrimental effects, causing pathological cardiac hypertrophy or increasing the tumorigenesis risk, especially for the global genetically modified animal models.^199–201^ Although some pharmacological approaches using IGF1 have shown protective effects against cardiac injury and dysfunction in animal experimental studies,^202,203^ clinical trials reported that chronic administration of IGF1 to human participants did not confer significant cardioprotective effects and may even cause cardiac fibrosis.^204^ Thus, other strategies that may activate the IGF1/PI3K/AKT in the heart require further investigation.^205–207^ Gene therapy targeting PI3K may also be potential strategy for protecting against cardiac remodeling in diabetic cardiomyopathy and in pressure overload-induced pathological hypertrophy model as well.^119,208^ In addition to the IGF1/PI3K/AKT pathway, other signaling molecules have been studied in the exercised heart. The use of inducible, cardiac-specific genetically modified animal models facilitates the functional studies of specific exercise-regulated molecules and the investigation of their therapeutic effects after the onset of CVD.^128,129^

Gene therapy has been widely used in animal experimental models of CVD.^209^ Different viral vectors are available for delivering targeted genes in vivo. Adenovirus-based gene delivery has robust transgene expression and high transduction rate, however, its application may be limited by its short-term effect and non-specific gene delivery. The lentivirus vector facilitates long-term gene expression in vivo at the expense of genomic integration. In comparison, the adeno-associated viruses (AAVs) are promising vectors for both long-term and tissue/cell-specific gene expressions.^210^ Among different AAV serotypes, AAV9 and AAV6 vectors are mostly used for gene expression in the study of cardiovascular system due to their cardiotropism advantage.^208,211,212^ The AAV9 or AAV6 mediated gene expression with a cardiac muscle cell-specific promoter such as α-myosin heavy chain (α-MHC) and cardiac Troponin T (cTnT) will further enhance the cardiotropic delivery.^213^ AAV-mediated gene therapy and its implications in CVD has been reviewed in detail previously.^209,214^ In a murine model of diabetic cardiomyopathy, a single tail vein injection of recombinant AAV6 (rAAV6)-caPI3K gene therapy was efficient to attenuate myocardial hypertrophy, cardiac fibrosis, and cardiac dysfunction.^208^ The delivery of rAAV6-caPI3K was applied after the manifestation of diabetic cardiomyopathy, suggesting a therapeutic potential of activating PI3K in the treatment of cardiac remodeling in diabetes.^208^ In another murine model of myocardial I/R injury, a therapeutic delivery of AAV9-miR-486 within 30 min after I/R injury showed beneficial effect in reducing myocardial apoptosis, cardiac remodeling, and cardiac dysfunction at both acute and chronic injury phase.^180^ The upregulation of miR-486 in the heart was found to be persistent even after 7 weeks post injection.^180^ For targeting exercise-regulated lncRNA CPhar, a tail vein injection of AAV9-short hairpin (sh)-CPhar attenuated exercise-induced physiological cardiac hypertrophy, while injection of AAV9-CPhar was protective against cardiac remodeling at 3 weeks post myocardial I/R injury.^51^ The long-term gene expression has also been observed with AAV9-lncExACT1 driven by a cardiac-specific troponin promoter showing upregulated lncExACT1 even at 16 weeks post injection which induced pathological cardiac hypertrophy.^46^ Despite the growing progress in AAV-mediated cardiac gene therapy, challenges remain to overcome host neutralizing anti-AAV antibodies, AAV delivery methods, and large-scale manufacture of AAV which will facilitate AAV cardiac gene therapy in large animal models and promote the translation to clinical application in the future.^210^

In addition, oligonucleotide-based gene therapy using miR mimic or agomiR, noncoding RNA inhibitors (antisense oligonucleotides, locked nucleic acid, antagomiR), small interfering RNA, and modified RNA, has also been applied to regulate gene expressions in vivo. These oligonucleotides can be commercially synthetized and are easy to dose in vivo.^214^ To investigate the effect of reducing lncExACT1 in physiological cardiac hypertrophy and in the protection against cardiac remodeling, a tail vein injection of the LNA antisense oligonucleotide (GapmeR) specific to lncExACT1 led to reduced lncExACT1 expression which induced physiological cardiac hypertrophy and protected against cardiac remodeling and heart failure.^46^ Currently, the viral vector- and oligonucleotide-based gene therapies are recognized as prosperous therapeutic strategies explored in pre-clinical experiments and clinical trials.^215^ Nonetheless, gene therapy is still in infancy and further investigation of efficacy and safety is required for those targets that have shown promising experimental results in the treatment of CVD.

**Fig. 7:**
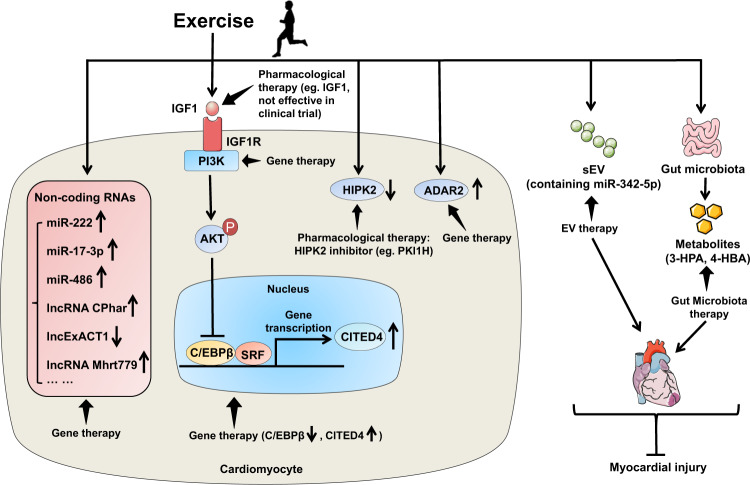
A schematic diagram of the potential therapeutic strategies based on exercise-regulated signaling pathways. Pharmacological therapy and gene therapy can target exercise-regulated signaling pathways (e.g., IGF1/PI3K/AKT, C/EBPβ/CITED4, noncoding RNAs, HIPK2, and ADAR2) to exert cardioprotective effect. Meanwhile, exercise-regulated small extracellular vesicles and gut microbiota as well as related metabolites can be applied to be potential therapeutic strategies for myocardial injury. IGF1 insulin growth factor 1, IGFR1 insulin growth factor 1 receptor, PI3K phosphoinositide-3 kinase, AKT protein kinase B, C/EBPβ CCAAT/enhancer binding protein β, SRF serum response factor, CITED4 CBP/p300-interacting transactivator with ED-rich carboxyterminal domain 4, HIPK2 homeodomain-interacting protein kinase 2, sEV small extracellular vesicles, 3-HPA 3-hydroxyphenylacetic acid, 4-HBA 4-hydroxybenzoic acid

Since exercise training can modify the number and cargo of extracellular vesicles which could partly mediate exercise’s benefits for the cardiovascular health, extracellular vesicle-based therapies prompt further investigation.^25,26,97,181^ In addition, exercise-regulated microbiota and related metabolites could be applicated to protect against myocardial infarction.^101^ A schematic diagram of the potential therapeutic strategies based on exercise-regulated signaling pathways is presented in Fig. 7.

## Multiple factors influencing the effect of exercise in cardiovascular health

The current knowledge and optimal design of animal exercise studies have been reviewed in detail previously which is of great importance to guide high quality-controlled animal exercise studies that will lead to a deeper understanding of exercise-associated cardioprotection.^21,216^ It is noteworthy that there are multiple factors that may influence the adaptations and outcomes of exercise.

### Genetic background

In animal experimental exercise studies, genetic background or molecular modifications may cause impaired exercise capacity.^217^ An increase of citrate synthase activity upon exercise as measured in mixed skeletal muscles including tibialis anterior and gastrocnemius helps to compare whether the animals had comparable exercise capacity among different groups.^110^ Also, whether genetic differences of human individuals influence their exercise habitude or cardiovascular responses upon exercise remain to be clarified.

### Sex difference

Sex difference may also influence the effect of exercise.^218^ It has been demonstrated that female mice developed greater increase in physiological myocardial hypertrophy after treadmill or voluntary wheel running.^219^ Moreover, although both male and female mice showed an increase in left ventricular mass after treadmill running, male and female mice had different regulation in Akt and MAPK signaling pathways in response to exercise.^220^ Further study is required to determine sex-specific signaling pathway responses upon exercise.^221^

### Exercise protocol or program

The exercise modality, intensity, frequency, and duration need to be further studied in both animal exercise models and clinical cohort studies.^222^ Currently, treadmill running, wheel running, and swimming exercise are the most widely used animal exercise models in the study of CVD, facilitating the standardization, comparison, and promotion of exercise studies from different research groups worldwide.^21,216^ However, exercise intensity and frequency such as interval high-intensity treadmill running *versus* moderate-intensity continuous treadmill running can have different cardiovascular responses by differentially regulating signaling pathways.^223^ It has been reported that high-intensity exercise in hypoxia is more effective to improve vascular function through enhanced NO bioavailability.^223^ Future work is needed to compare the efficacy of different exercise training protocols on cardiovascular responses and to clarify the signaling pathways involved.

For humans, the World Health Organization 2020 guidelines recommended that all adults should undertake 150–300 min of moderate-intensity or 75–150 min of vigorous-intensity physical activity, or an equivalent combination of both moderate- and vigorous-intensity aerobic physical activity per week.^16^ However, for CVD patients, low-to-moderate intensity of aerobic exercise is recommended to stable patients with NYHA class I-III; low intensity (<40% of VO_2peak_) is recommended in NYHA class III patients.^224,225^ Resistance training can be implemented after 3 weeks of aerobic exercise in CVD patients with the assessment of muscle strength test. However, the indications can vary and be modified according to the functional class of patients which depend on cardiopulmonary exercise testing, 6-min walk test, echocardiography, and biochemical analyses by the doctors.

In addition to different exercise training protocols or programs, the molecules and signaling pathways that are regulated by exercise may have the value to reflect the effect of exercise in cardiovascular healthy in humans. As the key regulators of exercise-induced physiological cardiac hypertrophy, miR-222 and miR-17–3p were found to be markedly upregulated in serum from heart failure patients after a cardiopulmonary exercise testing.^47,186^ Compared to healthy controls, serum level of miR-222 was significantly lower in patients with myocardial infarction at hospitalization.^188^ Moreover, lower miR-222 level was associated with rehospitalization or death during 1-year follow-up in these patients, serving as a potential biomarker for poor prognosis of myocardial infarction.^188^ Indeed, it is worth exploring the values of exercise-regulated molecules such as noncoding RNAs serving as potential biomarkers that can indicate exercise efficiency or CVD prognosis.

### Exercise intolerance

Exercise intolerance is a crucial problem in patients with heart failure, which is a hallmark of poor prognosis. Among the different methods used to assess exercise and functional capacity (e.g., NYHA functional classification, 6-min walk test, graded exercise testing with electrocardiography (ECG), and cardiopulmonary exercise testing), cardiopulmonary exercise testing is golden standard. Exercise intolerance in patients with heart failure can be attributed not only to reduced cardiac reserve and pulmonary reserve, but also to skeletal muscle dysfunction and other factors such as peripheral vascular dysfunction, obesity, and nutritional factors.^226^ Thus, in addition to the beneficial effect of exercise to the myocardium, it is also important to elucidate the function and underlying mechanism of exercise in the extracardiac tissues and organs. For example, pathological cardiac stress usually causes cardiac hypertrophy but leads to atrophy of skeletal muscles. Heart failure also induces mitochondria dysfunction of skeletal muscles.^227^ While exercise increases myofibrillar protein synthesis and the number of satellite cells and myonuclei, and increases capillary density and mitochondrial network in skeletal muscles as well. Endurance exercise leads to a switch to oxidative muscle fibers (type I), while resistance exercise leads to a switch to glycolytic muscle fibers (type II) and induces robust muscle hypertrophy.^228^ The Akt/mTOR, AMPK, PGC1α, and Ca^2+^/calmodulin-dependent protein kinase signaling pathways are involved in exercise-induced hypertrophy and mitochondrial adaptations of skeletal muscle.^228^ In addition to the skeletal muscle, the beneficial effect of exercise on other peripheral factors such as peripheral vascular dysfunction, obesity, and nutritional factors deserve further investigation. It is important to recognize that distinct causes and comorbid conditions may coexist which contribute to the pathophysiology of exercise intolerance in patients with heart failure, a combined pharmacological and nonpharmacological intervention (e.g., exercise training, diet, etc.) are required to improve exercise capacity and prognosis of patients.^226^

### Possible adverse effects of exercise

In addition to investigate the beneficial effect of exercise in cardiovascular health, the safety of exercise should also be evaluated aiming to avoid any possible adverse effects caused by excessive exercise. Compared to exercise-induced physiological cardiac hypertrophy, intensive exercise training was found to induce pathological alterations in the cardiovascular system at functional, structural, and molecular levels. After 16 weeks of vigorous running, rats developed eccentric hypertrophy and ventricular dilatation, together with diastolic dysfunction and increased arrhythmia inducibility.^229^ Meanwhile, significant cardiac fibrosis was detected in the atriums and right ventricle but not in the left ventricle of intensively-exercised rats accompanied with increased pro-fibrotic gene and protein expressions; these fibrotic changes were reversible after several weeks of exercise cessation.^229^ Moreover, treatment with losartan, an angiotensin II type 1 receptor antagonist, was able to alleviate cardiac fibrosis induced by intensive exercise.^230^ In humans, plasma levels of carboxyterminal propeptide of collagen type I (PICP), carboxyterminal telopeptide of collagen type I (CITP), and tissue inhibitor of matrix metalloproteinase type I (TIMP-1) were higher in veteran athletes compared to sedentary individuals, providing biochemical evidence of disruption of collagen equilibrium in veteran athletes.^231^ Indeed, in addition to cardiac morphological and functional measurements, biomarkers that can be measured easily in human blood samples are required to be identified which will be useful to predict the intolerance of exercise or the risk of adverse effect of excessive exercise in patients.

## Conclusion and future prospects

Exercise training has been widely recognized as a healthy life style modification as well as an effective non-drug therapeutic strategy for CVD. Both in vitro and in vivo studies have shed light to the protective effects and mechanisms of exercise against cardiovascular injury.^232^ In addition to the preventive benefits of exercise, its therapeutic effects after the onset of CVD have also been observed and established.^188^ An increasing number of exercise-regulated molecules and signaling pathways have been identified, which provides potential therapeutic targets for CVD. Compared to small molecule-based therapy, gene therapy such as viral vector-based therapies (e.g., adenovirus, lentivirus, AAV) and oligonucleotide-based therapies (e.g., mRNA, miRNA mimics, antisense oligonucleotides, siRNA) provide more interventional targets available that were, however, previously “undruggable” by small molecules.^233^ More investigation is required to evaluate the protective effect of gene therapy targeting these exercise-regulated signaling pathways and noncoding RNAs in CVD. Despite the rapid and cost-effective development, the delivery system of gene therapy needs further exploration for more specific targeting, more effective therapy, and lower immunogenicity.^233^ Very importantly, the therapeutic effects based on exercise-regulated molecules and signaling pathways need to be evaluated from small animal models to large animal models, and further to be translated for treatment of CVD in humans.^181^ Mechanistically, the intricate communication network between other organs or tissues (e.g., skeletal muscles, lungs, gut microbiota, brown adipose tissue, etc.) and the heart, as well as the effects of exercise on this cross-talk, warrants further investigation.^25–27^ High-throughput and multi-omics technologies and integrated analysis will be powerful tools to identify valuable exercise-responsive factors and mediators of communication between the heart and other tissues/organs.^234–236^ The study of the exercise-induced epigenetic modifications in special populations such as CVD patients or in the elderly will also advance our knowledge on exercise-induced beneficial cardiovascular adaptations.^237,238^ It would also be of great importance to identify the exercise-regulated molecules as potential biomarkers for guiding a more effective and safer exercise training for CVD patients, and predicting the prognosis of patients as well. Collectively, the data presented in this review suggest that an in-depth understanding of the signaling pathways and mechanisms involved in exercise’s benefits for cardiovascular health will promote the identification and guide the development of novel therapeutic targets and strategies to combat CVD in the future.
